# Anterior cruciate ligament—return to sport after injury scale: validation of the Norwegian language version

**DOI:** 10.1007/s00167-020-05901-0

**Published:** 2020-02-15

**Authors:** Anne Gro Heyn Faleide, Eivind Inderhaug, Willemijn Vervaat, Kyrre Breivik, Bård Erik Bogen, Ingunn Fleten Mo, Ingrid Trøan, Torbjørn Strand, Liv Heide Magnussen

**Affiliations:** 1 Haraldsplass Deaconess Hospital, Ulriksdal 8, 5009 Bergen, Norway; 2 The University of Bergen, Bergen, Norway; 3 Haukeland University Hospital, Bergen, Norway; 4 NORCE Norwegian Research Centre, Bergen, Norway; 5 Western Norway University of Applied Science, Bergen, Norway; 6 Oslo University Hospital, Oslo, Norway

**Keywords:** ACL-RSI, ACL reconstruction, Return to sports, Psychological response, Psychological readiness, Fear of injury

## Abstract

**Purpose:**

Evidence is emerging on the importance of psychological readiness to return to sport after anterior cruciate ligament (ACL) reconstruction. The ACL-Return to Sport after Injury scale (ACL-RSI) is developed to assess this. The aim of the current study was to translate ACL-RSI into Norwegian and examine the measurement properties of the Norwegian version (ACL-RSI-No).

**Methods:**

ACL-RSI was translated according to international guidelines. A cohort of 197 ACL-reconstructed patients completed ACL-RSI-No and related questionnaires nine months post-surgery. One hundred and forty-six patients completed hop tests and 142 patients completed strength tests. Face and structural validity (confirmative factor analysis and explorative analyses), internal consistency [Cronbach’s alpha (*α*)], test–retest reliability [Intraclass Correlation Coefficients (ICC)], measurement error [Standard error of measurement (SEM) and smallest detectable change at individual (SDC_ind_) and group level (SDC_group_)] and construct validity (hypotheses testing; independent *t* tests, Pearson’s *r*) were examined**.**

**Results:**

ACL-RSI-No had good face validity. Factor analyses suggested that the use of a sum score is reasonable. Internal consistency and test–retest reliability were good (α 0.95, ICC 0.94 (95% CI 0.84–0.97) and measurement error low (SEM 5.7). SDC_ind_ was 15.8 points and SDC_group_ was 2.0. Six of seven hypotheses were confirmed.

**Conclusions:**

ACL-RSI-No displayed good measurement properties. Factor analyses suggested one underlying explanatory factor for “psychological readiness”—supporting the use of a single sum score. ACL-RSI-No can be used in the evaluation of psychological readiness to return to sport after ACL injury.

**Level of evidence:**

III.

## Introduction

A majority of patients with an anterior cruciate ligament (ACL) tear choose to undergo surgery since their aim is to return to pre-injury level of sports [1, 2]. Recent research brings daunting news for these patients as up to 30% are reported to experience recurrent instability or a new ACL injury in the contralateral knee [3, 4]. In spite of stabilizing surgery and extensive postoperative rehabilitation, up to 40% of patients fail to return to their pre-injury level of sports and less than half return to competitive sport [1, 5].

Rehabilitation after ACL reconstruction (ACLR) has been focused on identifying, measuring and treating physical factors like muscle strength and neuromuscular function [6]. Over the past decade, several reports have displayed how fear of re-injury is a common reason for changing or ceasing sports participation—thereby increasing the focus on psychological responses [1, 5]. The term “psychological readiness” is frequently used to describe mental factors influencing return to sports (RTS) after ACL injury. These factors include realistic expectations, confidence in performance, high levels of self-efficacy and low levels of fear and anxiety [6].

Low fear of re-injury and high “psychological readiness” have been found to favor a return to pre-injury level of sport [1, 5, 7]. It is not necessarily desirable for the patients to remove fear completely, as some reservation may be protective in the gradual return to vigorous activity [6]. Nevertheless, if patients make well-informed choices aiming to RTS, assessing psychological readiness can aid clinicians in identifying patients who are inhibited by inexpedient mental responses. Hopefully, early detection can lead to proper interventions in a joint effort towards reaching the athletes’ goals.

The Anterior Cruciate Ligament-Return to Sport after Injury (ACL-RSI) scale was developed with the aim of identifying patients who may struggle with the resumption of sports [7]. The questionnaire covers key aspects of psychological readiness for RTS including emotions (e.g. fear and frustration), confidence in performance and risk appraisal [7]. These aspects are hypothesized to be intimately related and evidence for one common construct, named “psychological readiness”, exists. This means that one underlying construct account for most of the variance in scores on the ACL-RSI—therefore, the use of one single sum score on the scale can be justified [2, 7–10]. The ACL-RSI has several translations all reported to have adequate to good measurement properties [2, 9, 11–14]. Currently, no Norwegian translation of the scale exist.

Previous evidence on structural validity of ACL-RSI has been based on principal component analysis (PCA) [2, 7, 9, 10]. In the current study, a confirmative factor analysis (CFA) was planned as this has not been performed on the ACL-RSI previously. CFA is highly recommended when a predetermined hypothesis on the construct exist [15, p. 72]. The hypothesis was that a Norwegian version of ACL-RSI (ACL-RSI-No) would be valid and reliable—and that one common construct (psychological readiness) for all items of ACL-RSI could be confirmed (one-factor solution). The aim of the present study was to provide Norwegian clinicians with a tool to pinpoint patients who may struggle with RTS and, further expand knowledge on validity of the ACL-RSI by translating the scale from English to Norwegian and examine face and structural validity, internal consistency, test–retest reliability, measurement error and construct validity.

## Materials and methods

The study was approved by the NSD (Norwegian Centre for Research Data) Data Protection Official for Research, project number 44708 and the Regional Committee for Medical and Health Research Ethics West 2015/1159.

Patients who had undergone ACLR at three Norwegian Orthopedic Centers were recruited from 2015 to 2018. They were eligible for participation if ≥ 16 years at the time of follow-up, fluent in Norwegian and had engaged in physical activity or sports. Patients with concomitant posterior cruciate ligament injury were excluded. All patients were asked to give their written, informed consent.

**Fig. 1:**
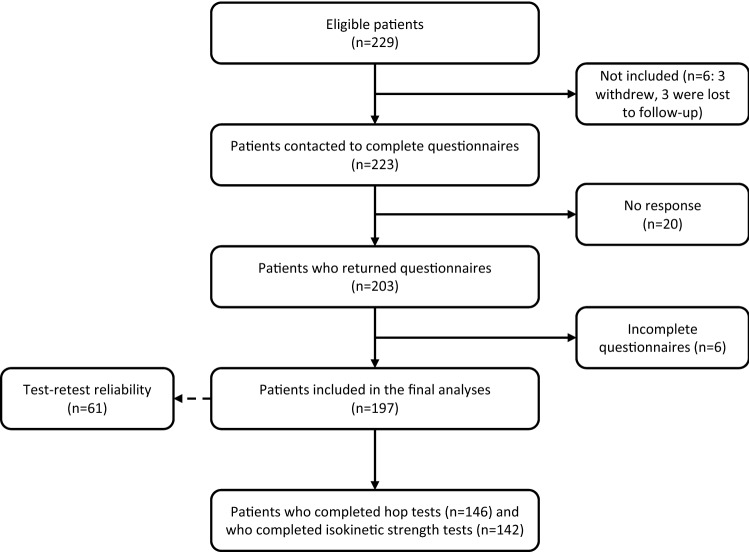
Flowchart of patients’ participation

Two hundred and twenty-nine patients met the inclusion criteria and all of these volunteered for the study (see Fig. 1 for flowchart and Tables 1 and 2 for demographic data and descriptive statistics on the measurements).

**Table 1:** Baseline patient characteristics, including pre-injury activity/sport level and main types of activity/sports performed (*N* = 197)

Characteristics	
Age, mean (SD), min–max	29.5 (9.7), 16–53
Gender, *N* (%), men	107 (54)
Months after ACLR, mean (SD), min–max	11 (2.0), 7.8–20.6
Hamstrings tendon graft, *n* (%)	64 (33)
Patellar tendon graft, *n* (%)	115 (59)
Quadriceps tendon graft, *n* (%)	6 (3)
Revisions, *n* (%)	12 (6)
Preinjury level of activity/sport	
Elite, *n* (%)	13 (7)
Medium to high level of competition, *n* (%)	59 (30)
Low level of competition, *n* (%)	64 (32)
Recreational level, *n* (%)	61 (31)
Three main primary activities/sports	
Soccer, *n* (%)	94 (48)
Alpine skiing, *n* (%)	21 (11)
Handball, *n* (%)	19 (10)

**Table 2:** Descriptive statistics on measurements used in hypothesis testing (*N* = 197)

Measurements	Mean (SD), min–max	Pearson’s *r*	*p*-value
ACL-RSI	55.7 (23), 0–100		
IKDC 2000	78.7 (13.2), 26.4–100	0.61	< 0.01
TSK^a^	24.3 (6.1), 13–47	− 0.34	< 0.01
KOOS pain	89.4 (9.9), 44–100	0.48	< 0.01
KOOS symptoms	83.4 (12.5), 43–100	0.37	< 0.01
KOOS function in daily living	95.9 (7.4), 54–100	0.43	< 0.01
KOOS function in sport and recreation^b^	73.7 (19.6), 5–100	0.49	< 0.01
KOOS knee-related Quality of Life	64.7 (18.1), 6–100	0.66	< 0.01
Hop test, LSI %^c^	95.5 (9.2), 44.8–112.4	0.28	< 0.01
PT extension 60°/s LSI %^d^	− 17.7 (14.8), − 60.2 to 30.5	0.17	0.04
PT flexion 60°/s LSI %	− 4.3 (17.5), − 47.4 to 49.5	0.14	n.s
TW extension 60°/s LSI %	− 11.6 (15.6), − 59.6 to 42.5	0.13	n.s
TW flexion 60°/s LSI %	2.9 (28.3), − 56.7 to 109.7	0.10	n.s

Includes correlations (Pearson’s *r*) between nine-month follow-up scores on ACL-RSI-No, and measures of fear of movement and function

*ACL-RSI* Anterior Cruciate Ligament-Return to Sports after Injury Scale, *IKDC 2000* The International Knee Documentation Committee Subjective Knee Form 2000, *TSK* Tampa Scale of Kinesiophobia, *KOOS* The Knee injury and Osteoarthritis Outcome Score, *LSI* Leg Symmetry Index, *PT* peak torque, *TW* Total Work

^a^1 missing questionnaire in TSK

^b^2 missing questionnaires in KOOS subscales Sport and recreation and Quality of Life

^c^146 patients completed hop tests

^d^142 patients completed isokinetic strength tests, invalid results for flexion in three of these

### Translation and cross-cultural adaptation

ACL-RSI was translated and cross-culturally adapted into Norwegian applying the guidelines described by Beaton and colleagues involving the author of the original scale [16]. As part of this work, an expert committee consisting of two researchers experienced in questionnaire translation, six health professionals (three physiotherapists specializing in orthopedic physiotherapy, two orthopedic surgeons, one psychologist) and two language professionals were established. Five patients who had undergone ACLR completed the questionnaire and were interviewed about their interpretation of questions and potential ambiguities in wording. Face validity and cultural adaptation of the Norwegian version were assessed by both the expert committee and testers of the pre-final version.

### Test procedure

Participants completed a battery of questionnaires nine to twelve months after surgery—the point where many consider RTS [1]. In one of the centers (recruiting the majority of patients), patients underwent functional testing (single-leg hop tests and isokinetic strength tests) for assessment of readiness to RTS after questionnaire completion. Patients recruited from the two other centers received questionnaires by mail.

The *ACL-RSI* comprises 12 questions where patients grade their answers on a Likert scale ranging from zero to 100 with ten-point increments [7]. Higher scores indicate greater psychological readiness towards RTS [10]. The *International Knee Documentation Committee Subjective Knee Form (IKDC) 2000* measures symptoms, function and sports activity in a variety of knee conditions (including ligament surgeries) with score range from zero (low function) to 100 (high function) [17]. The *Tampa Scale of Kinesiophobia (TSK)* measures fear of movement in patients with low back pain [18] but has also been used to examine fear of re-injury in patients with ACL injuries [19]. The *Knee injury and Osteoarthritis Outcome Score (KOOS)* was developed for patients with knee injuries and/or osteoarthritis and is frequently used in patients after ACLR. It comprises five domains: pain, other symptoms, function in daily living, function in sports and recreational activities and quality of life (QoL) [20]. Total score of each subscale ranges from zero to 100 where a higher score indicates good function [21]. A custom-made questionnaire included questions about the surgery, previous injuries/surgeries, type and level of activity/sport performed before injury and status on RTS after ACLR. Level of participation was categorized as elite level, medium/high level of competition, low level of competition and recreational level.

The single-leg hop tests comprise four tasks: a single hop for distance (centimeters (cm)), a triple hop for distance (cm), a six-meter timed hop (seconds (sec)) and a triple crossover hop for distance (cm). Results are presented as a percentage difference between the performance of the limbs (Leg Symmetry Index, LSI %) for each test individually and as a sum score where all four tests are combined. The hop tests are reliable and valid performance tests for patients undergoing rehabilitation after ACLR, with reported test–retest Intra-class Correlation Coefficient (ICC) of 0.93 and Standard error of measurement (SEM) 3.0 for the sum score of all four tests [22, 23].

Isokinetic strength testing of knee flexion and extension was performed at 60°/sec (five repetitions) angular velocity using a dynamometer system (Biodex system 3 dynamometer, Biodex Medical Systems Inc., Shirley, New York). Performance is reported as an LSI (%) in peak torque (Newton-meters, Nm) and total work (Watt, W). Isokinetic strength testing is reliable (test–retest ICCs for peak torque and total work > 0.90) and considered to be the gold standard performance measure in ACL rehabilitation [24, 25].

### Examination of measurement properties

The Consensus-based Standards for the selection of health Measurement Instruments (COSMIN) were applied [26, 27]. These guidelines provide definitions and criteria for evaluation of the quality of a questionnaire’s measurement properties.

For evaluation of *structural validity*, CFA was performed to examine whether the proposed one-factor solution (psychological readiness) had a good fit to the data. Descriptive goodness-of-fit indices were used: Chi square, standardized root mean square residual (SRMR), root mean square error of approximation (RMSEA) and comparative fit index (CFI) [28, pp. 67–73]. The recommended criteria for good fit of a model are CFI close to or higher than 0.95, SRMR close to or lower than 0.08 and RMSEA close to or lower than 0.06 [29]. If a poor fit was found, explorative analyses would be applied to determine whether the scale was unidimensional enough to be treated as such or if more factors were needed to model the item responses [30].

*Internal consistency* was assessed by Cronbach’s alpha coefficient (*α*): 0.70 is acceptable, 0.80 is preferable and > 0.95 might indicate item redundancy [27]. *Test–retest reliability* was examined in a subgroup of 61 patients—1 week prior to and again at the start of the follow-up evaluation. Two-way random ICC_2.1_ for relative reliability was calculated [27]. The ICC should be at least 0.70 (0.70–0.89 indicate high correlation, 0.90–1.00 indicate very high correlation) [15, p. 120]. To establish absolute reliability, which is an expression of the *measurement error*, SEM was calculated from the mean of the variances between tests [27]. A 95% Confidence Interval (CI) of SEM was made to suggest the limits of measurement error (1.96*SEM). Based on SEM, smallest detectable change at individual level (SDC_ind_) was calculated (1.96 × √2 × SEM), reflecting the smallest change score that with *P* < 0.05 can be interpreted as real change, not measurement error. The SDC for a group of persons (SDC_group_) was calculated (SDC_ind_/√n) [27]. Limits of Agreement (LoA) was evaluated using a Bland–Altman plot [15, p. 113].

*Construct validity* with hypothesis testing is recommended when there is no gold standard to compare the scores on the measurement instrument to [15, p. 169]. Pre-defined hypotheses were formed based on validation studies of ACL-RSI, studies on RTS after ACLR, findings from previous translations and clinical experience (for hypotheses, see Table 5). A disparity between performance on functional tests and RTS has been highlighted as a reason for focusing on psychological responses in ACL rehabilitation [1]. We, therefore, included hypotheses on associations between functional tests and ACL-RSI. Correlations were investigated using Pearson’s *r*; 0.10–0.29 were considered small, 0.30–0.49 medium and 0.50–1.0 large [31, pp. 79–81]. For discriminative ability, independent *t* tests were used.

The ACL-RSI-No as a whole and each individual item was examined for *floor and ceiling effects*. If more than 15% of the patients achieve the lowest or highest score possible on the scale, this suggests that floor or ceiling effects are present [32].

### Statistical analysis

IBM SPSS Statistics Version 24.0 software was used for descriptive statistics, testing of normality, the examination of internal consistency, test–retest reliability, Bland–Altman plot, hypothesis testing (significance level *P* < 0.05 for *t* tests) and floor and ceiling effects. For continuous variables, means and standard deviations (SD) are presented and for categorical variables absolute and relative frequencies are presented. CFA, scree plot and parallel analysis were performed using JASP (Version 0.9). Measurement error was calculated in Microsoft Excel 2010.

## Results

The expert committee and the five testers agreed that the ACL-RSI-No had good *face validity* with relevant content for the patient group at the time of administration. The questions were easy to understand and contained aspects of importance for RTS that were not covered in the other questionnaires. No special cultural adaptation was recommended.

**Fig. 2:**
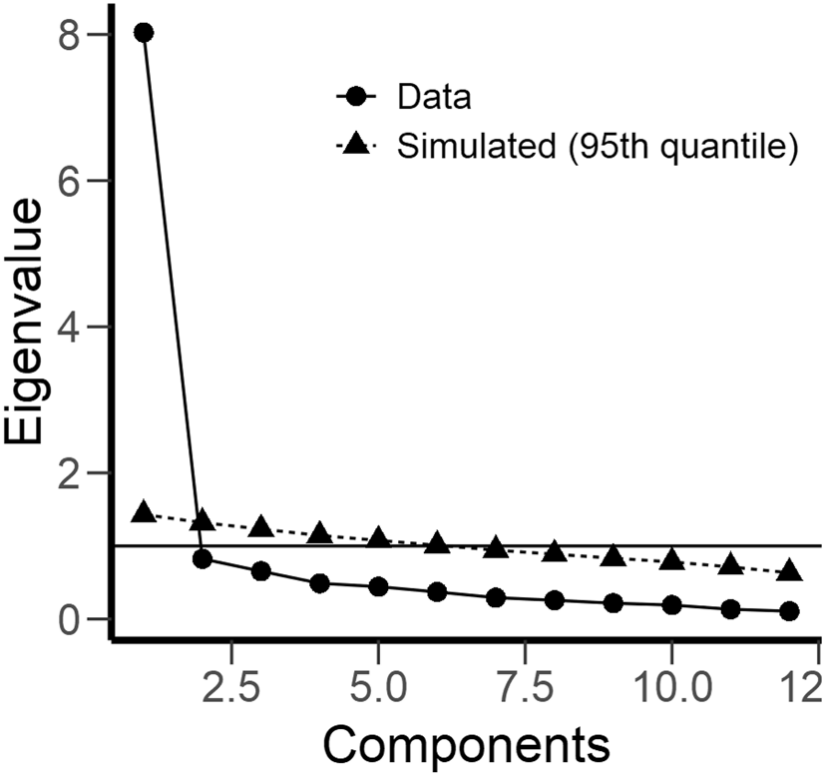
Scree plot and parallel analysis

Results from the *CFA* displayed that a one-factor solution had a poor fit to the data (Chi Square 274.80 (degrees of freedom (df) 54, *P* < 0.01), SRMR 0.05, RMSEA 0.14 (95% CI 0.13–0.16, *P* < 0.01) and CFI 0.90). Correlations between 15 pairs of residuals were needed to achieve a satisfactory fit by conventional standards and COSMIN criteria (*χ*^2^ 64.30 (df = 39, *P* < 0.01), SRMR 0.03, RMSEA 0.06 (95% CI 0.03–0.08, n.s. and CFI 0.99). Further explorative analyses were, therefore, conducted. These suggest that treating the scale as unidimensional is justified: The reliability of the one-factor solution (when the correlated error terms were accounted for) was high (0.93), which means that 93% of the variance of the scale is explained by true variance (the common factor). The size of the factor loadings in the one-factor CFA solution remained robust (none of the factor loadings changed more than 3%) regardless of whether the correlated error terms were included in the model or not. Inspection of scree plot and parallel analysis strongly indicate a one-factor solution (Fig. 2). The ratio between the two first eigenvalues was eight. A two-factor explorative factor analysis (EFA) was performed and correlation between the extracted factors was very high (0.85). This suggests lack of discriminative validity and further support the fact that item responses are determined by one dominant factor.

**Table 3:** ACL-RSI-No scores in returners and non-returners to pre-injury activity/sport and pre-injury level of activity/sport (*N* = 197)

	Yes Mean (SD)	No Mean (SD)	Mean difference	95% CI of difference	*p*-value
Return to same activity	68.0 (19.5) *n* = 95	44.2 (19.9) *n* = 102	23.9	18.3–29.4	< 0.01
Return to same level	70.6 (18.6) *n* = 48	50.8 (22.3) *n* = 149	19.8	12.8–26.8	< 0.01

*ACL-RSI* Anterior Cruciate Ligament-Return to Sports after Injury Scale

**Fig. 3:**
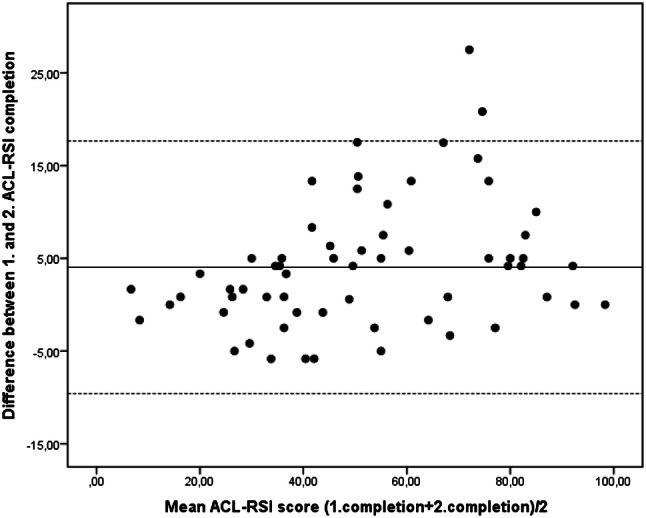
Bland Altman Plot displaying Limits of Agreement

*Internal consistency* (*α*) was 0.95 which is close to the model-based alpha derived from the CFA (0.93). *Test–retest reliability* was very high (Table 3). Measurement error (SEM) was 5.7 implicating that change in score for one individual needs to exceed 15.8 points and on group level 2.0 to be interpreted as true change (exceeding measurement error). For LoA, see Bland Altman Plot in (Fig. 3).

**Table 4:** Test re-test reliability of the ACL-RSI-No (*N* = 61)

ACL-RSI-No 1. administration, mean (SD)	49.6 (22.0)
ACL-RSI-No 2. administration, mean (SD)	53.8 (24.2)
Mean difference	4.2
ICC 2.1. (95% CI)	0.94 (0.84–0 .97)
SEM	5.7
1.96*SEM	11.2
SDC individual	15.8
SDC group	2.0

*ACL-RSI* Anterior Cruciate Ligament-Return to Sports after Injury Scale, *ICC* Intra-class Correlation Coefficient, *SEM* Standard Error of Measurement, *SDC* Smallest Detectable Change

Six of seven pre-formulated hypotheses were confirmed indicating good *construct validity* (Tables 2, 4 and 5). The hypothesis on a small correlation between ACL-RSI-No and isokinetic strength tests was not supported. A small, but statistically significant (*P* = 0.04) correlation was found between ACL-RSI-No and performance on extension peak torque LSI at 60°/s, but for the rest of the isokinetic strength tests no significant association was found.

**Table 5:** Pre-defined hypotheses, including the result of hypothesis testing: + hypothesis confirmed, − hypothesis not confirmed

1	Patients who have returned to *pre-injury activity or sport* (at any level) have a significantly higher score on the ACL-RSI-No than those who have not returned	+
2	Patients who have returned to *pre-injury level* have a significantly higher score on the ACL-RSI-No than those who have not returned	+
3	There is a medium to large correlation (0.30 < *r* < 0.60) between IKDC 2000 and ACL-RSI-No	+
4	There is a medium to large negative correlation (− 0.30 < *r* < − 1.0) between TSK and ACL-RSI-No	+
5	There is a medium correlation (0.30 < *r* < 0.49) between KOOS and ACL-RSI-No and a large correlation (0.50 < *r* < 1.0) between KOOS QoL and ACL-RSI-No	+
6	There is a small to medium correlation (0.10 < *r* < 0.49) between the hop test (LSI % averaged sum score of all four tests) and ACL-RSI-No	+
7	There is a small correlation (0.10 < *r* < 0.29) between the isokinetic strength tests and ACL-RSI-No	−

No *floor or ceiling effects* were found for the overall score (0.5% of the patients had the lowest possible score (zero) and 0.5% had the highest score (100)). 3% of patients had a sum score of 10 or less and 5% had a score of 90 or more. For each question, the percentage of patients who had the lowest possible score ranged from 2 to 17%. The percentage of patients who had the highest score on each item ranged from 3 to 20%. Mean score on the individual items varied between 41.2 (SD 31.3) and 64.3 (SD 27.6).

## Discussion

The most important finding of the present study was support for good validity and high reliability of the ACL-RSI-No. Six of seven hypotheses were confirmed providing evidence for good construct validity. In the factor analyses, support for a one-factor structure (psychological readiness) was found—justifying the current use of a single sum score (from 0 to 100) for the scale.

Support for an one-factor solution (psychological readiness to return to sport) has been found in previous studies using PCA, except for the Spanish version were two dimensions (confidence in performance and fear/insecurity) were found [2, 7, 9, 10, 33]. PCA is widely used but has limitations as it is a data reduction method computed without regard for latent variables [34]. In accordance with COSMIN recommendations, the current study, therefore, started with CFA to evaluate whether the items fit a one-factor solution [15, p. 169]. As the analysis indicated an inadequate fit, explorative analyses were applied to determine whether the scale is unidimensional enough to be treated as such [30]. Findings from these analyses suggest that it is probably most parsimonious to treat the scale as essentially unidimensional: The scree plot and parallel analysis displayed that the ratio between the two first eigenvalues was well above the recommended rule of thumb (which is three) for regarding a scale as essentially unidimensional [30]. In line with this finding, the single factor in the CFA explained as much as 93% of the variance in ACL-RSI-No scores. The two-factor solution (EFA) had poor discriminative ability and is not recommended [28, p. 146]. Item response data is seldom strictly unidimensional and it is well known that it can be determined by a strong common factor even when the fit of a one-factor solution does not meet the recommended criteria of good fit [35]. To our knowledge, the current study is the first to apply CFA in the investigation of ACL-RSI factor structure. More studies applying such methodology should, therefore, follow the current work.

The finding of high test–retest reliability is in line with previous results [2, 9, 11, 13, 14]. For this study, a week between completions was chosen to ensure that the questionnaire was not fresh in memory at second administration which is recommended by Terwee et al. [27]. The phenomenon of psychological readiness to RTS was expected to be relatively stable in this period.

In the current study, SDC was calculated, providing information on how much scores must change to be interpreted as change exceeding measurement error [27]. The SDC should be smaller than the amount of change that is considered *clinically meaningful* (Minimal Important Change, MIC) [21]. To allow for the evaluation of treatment or monitor changes in health status (longitudinal validity), the questionnaire should be able to detect changes over time [15, pp. 202–203]. In this study, MIC and longitudinal validity were not assessed. For the Dutch version of ACL-RSI, responsiveness has been found to be sufficient on group level but limited for individuals [36].

Support for good construct validity was found as six of seven pre-defined hypotheses were confirmed. Patients who returned to pre-injury activity scored significantly higher on ACL-RSI-No than patients who had not returned – indicating good discriminant validity of the scale. This finding is in line with previous studies [2, 7, 13]. The finding of medium to large associations between the ACL-RSI-No and the IKDC 2000 and KOOS also corresponds to results from other studies [2, 9, 11, 13, 14]. IKDC 2000 and KOOS assess constructs of symptoms, pain and function [17, 20]. We, therefore, hypothesized some association between low levels of symptoms/pain and higher levels of functioning and psychological readiness to RTS. Since fear of re-injury, confidence and emotions are not directly assessed in IKDC 2000 and KOOS, we did not expect large associations. For the current young and active population, it is reasonable to infer that the ability to return to an active lifestyle is intimately related to high QoL. This may explain the finding of a high correlation between ACL-RSI-No and KOOS QoL. A higher score on the TSK has been associated with not returning to sport and inferior self-reported function [37]. The TSK displayed a medium negative correlation with ACL-RSI-No. This is slightly different from others reporting medium to large negative correlations and may possibly be explained by the use of the 13-item version (the only Norwegian translation available) in the current study compared to the 17-item version the other studies [2, 9, 11, 13, 14, 38].

Psychological and physical readiness to RTS does not necessarily coincide [1]. Physical function and psychological aspects are quite different constructs. Still, if a patient experiences a stable and well-functioning knee this will probably affect the psychological responses. Others have found a weak correlation with isokinetic strength tests and hop tests [39]. Therefore, a small significant correlation between performance on functional tests and ACL-RSI-No score was expected. This was confirmed for hop tests, but not for strength tests (except for a small, significant association for extension peak torque) in the current study. These results support the clinical observation that patients may score poorly on the ACL-RSI while performing well on physical tests and vice versa. This is a critical finding since the use of physical tests—such as dynamometer testing or hop-testing—is at current a dominant approach in RTS assessment [6, 40]. Studies aiming to evaluate psychological responses as part of the RTS testing are, therefore, warranted.

The current population is comparable to the populations described in studies of the original version of ACL-RSI and other language translations. Most studies include both elite athletes and patients involved in recreational activities, but different methods for describing type and level of sport makes comparing activity level across the studies difficult [2, 7, 9, 11–14]. A difference between studies in the postoperative time for assessment (from six to 24 months) should be taken into consideration as it might affect comparability.

The prospective design and large number of participants included in analyses represent strengths of the current work. A thorough factor analysis, including exploration of associations between physical tests and psychological responses, adds new knowledge to this research field. Our motivation for validation of a Norwegian version the ACL-RSI was to nuance the assessment of readiness to RTS after ACLR. This assessment is commonly performed approximately nine months after surgery [41], therefore—validation of the questionnaire in the timeframe it is intended used, pose a further strength of the study.

In the examination of construct validity, the measurement properties of the related questionnaires are important [15, p. 174]. The IKDC is reported to be valid in patients with mixed knee pathologies and injuries, but evidence on validity in ACL injured patients is limited with reports of problems with structural validity and in distinguishing clinically relevant changes from measurement error [42–44]. KOOS has been criticised for not having adequate measurement properties for use in patients after ACLR [21, 45]. Limited information is available about the Norwegian versions of IKDC 2000, TSK and KOOS. A proper assessment of measurement properties of the Norwegian IKDC 2000 has not been performed, procedures for translating KOOS are not published and TSK was validated for patients with sciatica [38, 46]. Although the Norwegian versions of IKDC 2000, KOOS and TSK are in widespread use and are well accepted in clinical and research communities—limitations in the comparative use of these questionnaires should be acknowledged.

The current study adds to the growing evidence on the validity of the ACL-RSI and implies that clinicians need to use more than physical tests in their evaluation of readiness to RTS after ACLR. Norwegian clinicians are provided with a tool to evaluate psychological readiness during rehabilitation and in RTS assessment to complement the physical tests.

## Conclusions

The Norwegian version of ACL-RSI has adequate to good measurement properties and can, therefore, be applied for use in the evaluation of psychological readiness to return to sport after ACL injury.

## Abbreviations

ACL: Anterior Cruciate Ligament
ACLR: Anterior Cruciate Ligament reconstruction
ACL-RSI: Anterior Cruciate Ligament-Return to Sport after Injury (Scale)
ACL-RSI-No: Anterior Cruciate Ligament-Return to Sport after Injury (Scale)-Norwegian Version
CFA: Confirmatory factor analysis
CFI: Comparative fit index
CI: Confidence interval
Cm: Centimeters
COSMIN: Consensus-based Standards for the selection of health Measurement Instruments
DF: Degrees of freedom
EFA: Exploratory factor analysis
ICC: Intraclass correlation coefficient
IKDC 2000: International Knee Documentation Committee Subjective Knee Form 2000
KOOS: The Knee injury and Osteoarthritis Outcome Score
LoA: Limits of Agreement
LSI: Leg symmetry index
MIC: Minimal important change
Nm: Newton-meters
NSD: Norwegian Centre for Research Data
PCA: Principle component analysis
QoL: Quality of life
r: Pearson product moment correlation coefficient
RMSEA: Root mean square error of approximation
RTS: Return to sports
SD: Standard deviation
SDC: Smallest detectable change
SDC_ind_: Smallest detectable change at individual level
Sec: Seconds
SEM: Standard error of measurement
SRMR: Standardized root mean square residual
TSK: Tampa Scale of Kinesiophobia
W: Watt
